# Method of Avoiding the Warping or Springing of Metallic Plates in the Process of Soldering Full Sets of Teeth

**Published:** 1855-04

**Authors:** 


### Selected Articles.

### METHOD OF AVOIDING THE WARPING OR SPRINING OF METALLIC PLATES IN THE PROCESS OF SOLDERING FULL SETS OF TEETH.

The frequent and vexatious *failures* in this important operation, render it very desirable with the mechanical Dentist, to have a process easy of execution, by which these failures may be always avoided; and the following plan has been so long and so successfully tried as to render its entire success unquestionable.

When the piece of work is ready for soldering, take three parts of well washed sand and one part of calcined plaster, mixed together in a dry state and kept in a box for daily use, and wet them to the condition of a paste, with which the plate and its arrangement of teeth are to be covered in the following manner. Place a sufficient quantity of the above named paste upon a piece of paper on a flat surface, and set the piece of work upon it, pressing it down into the wet mass until the metal comes almost, but not quite in contact with the paper.

Then bring up the mortar against the fronts of the teeth and over their ends or points the thickness of one third of an inch, also upon the metal plate to the depth of half an inch, excepting where the solder is to flow. When this has been carefully done, in such manner that the teeth and plate are covered equally to the depth prescribed, take a strip of platina plate one fourth of an inch broad, and as thick as ordinary plate used for full sets, long enough to embrace the outer circumference of the mortar or mixture of plaster and sand, and adjust it in contact with the same. This platina band must be provided with three hooks of the same metal, one inch in length, and soldered to the band so that when bent they may rest upon the plaster that covers the end of the teeth. One of these hooks should be in the center, or front, and one within half an inch of each end of the band, and both the band and the hooks may be covered with a thin coating of the mortar to keep the whole in place. Let the mortar then be trimmed to a uniform thickness, in order that the heat used in soldering may be equally diffused as it approaches the plate and teeth. Let the plaster set perfectly, and the whole be ready to be placed upon a bed of charcoal in a soldering cup ready to be heated. In order that the heat may be applied both steadily and progressively, the bottom of the sheet-iron soldering cup should be perforated with holes like a cullender, each hole of the diameter of a large pea. Place this cup, containing the work to be soldered, over a fire of live coals, either anthracite or charcoal, and let the coal within the cup remain ignited over a gentle draft, until the work resting upon it is heated almost to redness, when the blast of the blow-pipe may be applied and the work completed. Suction sets heated in this manner will fit the mouth as exactly after soldering as before, providing all particulars of the process be honestly observed.

*Rationale* of the process. .I The small quantity of plaster employed, compared with the sand, avoids the great expansion and contraction which unavoidably takes place when much plaster is used, inasmuch as the water absorbed by the plaster, is all driven off by the heat.

II. The particles of pure sand, or silex, suffer very little expansion or contraction by the heat employed in soldering.

III. The *gradual application* of the heat, implied in the foregoing description, causes all parts of the metal to expand and contract together, without any violent effect upon any particular part.

IV. The *equable manner* in which the mortar is spread on the surface of the metallic plate, has some influence on the fortunate result.

V. The *purity of the sand*, or the absence of all kinds of foreign matter, is essential to the perfect success of this process,—therefore, wash the sand.

The above is much the same as the plan recommended by Dr. Dwinelle to us last summer; we have not as yet tested it, relying more especially on a fine gold plate for suction operations, using solder which flows easily. We have always thought that solder which requires near the same amount of heat to flow it, as the plate itself, would always endanger the springing of the plate in the operation of soldering. We take especial pains to well anneal our plates after they are adjusted, and when this is done and the solder flows readily at a much less heat than is requisite to fuse the plate—we do not expect our plate to warp.

